# Natriuretic Peptides—New Targets for Neurocontrol of Blood Pressure via Baroreflex Afferent Pathway

**DOI:** 10.3390/ijms232113619

**Published:** 2022-11-07

**Authors:** Xinyu Li, Yali Cui, Qing Zhang, Qingyuan Li, Mengxing Cheng, Jie Sun, Changpeng Cui, Xiongxiong Fan, Baiyan Li

**Affiliations:** State-Province Key Laboratories of Biomedicine-Pharmaceutics of China, Key Laboratory of Cardiovascular Research, Ministry of Education, Department of Pharmacology, College of Pharmacy, Harbin Medical University, Harbin 150081, China

**Keywords:** natriuretic peptide, blood pressure regulation, baroreflex afferent function, high-fructose induced hypertension, gender difference

## Abstract

Natriuretic peptides (NPs) induce vasodilation, natriuresis, and diuresis, counteract the renin–angiotensin–aldosterone system and autonomic nervous system, and are key regulators of cardiovascular volume and pressure homeostasis. Baroreflex afferent pathway is an important reflex loop in the neuroregulation of blood pressure (BP), including nodose ganglion (NG) and nucleus tractus solitarius (NTS). Dysfunction of baroreflex would lead to various hypertensions. Here, we carried out functional experiments to explore the effects of NPs on baroreflex afferent function. Under physiological and hypertensive condition (high-fructose drinking-induced hypertension, HFD), BP was reduced by NPs through NG microinjection and baroreflex sensitivity (BRS) was enhanced via acute intravenous NPs injection. These anti-hypertensive effects were more obvious in female rats with the higher expression of NPs and its receptor A/B (NPRA/NPRB) and lower expression of its receptor C (NPRC). However, these effects were not as obvious as those in HFD rats compared with the same gender control group, which is likely to be explained by the abnormal expression of NPs and NPRs in the hypertensive condition. Our data provide additional evidence showing that NPs play a crucial role in neurocontrol of BP regulation via baroreflex afferent function and may be potential targets for clinical management of metabolic-related hypertension.

## 1. Introduction

Natriuretic peptides (NPs) are group of cardiovascular regulatory peptides involved in maintaining the body’s water and salt balance, and holding the homeostasis of the cardiovascular and renal system [1]. Its main members are atrial natriuretic peptide (ANP) [2], brain natriuretic peptide (BNP) [3], and C-type natriuretic peptide (CNP) [4], which are mainly produced in the atrium, ventricles of the heart, and the endothelium cells, respectively [5]. These peptides primarily function as endogenous ligands and mainly act via their membrane receptors including natriuretic peptide receptor A (NPRA), receptor B (NPRB), and receptor C (NPRC) [6]. NPRA/NPRB belongs to guanylyl cyclase (GC) and mediated the most physiological effects of NPs. NPRC is a disulfide-linked homodimer that is homologous to the extracellular domains of NPRA/NPRB and plays a primary role in the clearance of NPs from the extracellular environment via a receptor-mediated internalization and degradation processes [7].

The protective effects of NPs on cardiovascular function have been well identified, which include increasing the glomerular filtration rate, reducing aldosterone secretion, relaxing vascular smooth muscle in both arterioles and venules, suppressing the renin-angiotensin-aldosterone system (RAAS) and sympathetic nervous system (SNS) activity, inhibiting cardiac hypertrophy, and so on [8,9]. Moreover, NPs have been stated to play an intricate role in various metabolic processes, such as glucose homeostasis (increase circulating insulin and inhibit glucagon secretion) [10], fat metabolism (lipid mobilization in human white adipose tissue (AT), energy dissipation in brown AT, browning of white AT) [11], nutrient absorption, as well as food intake [12]. The mechanism of lipolytic action involves activation of NPRA, cyclic guanosine monophosphate (cGMP) signaling, and trans-location of hormone-sensitive lipase. Moreover, NPs enhance whole body energy expenditure via an upregulation of the uncoupling protein-1 (UCP1) in brown fat and activate mitochondrial biogenesis and uncoupling in white fat. In addition, NPRA knockout exhibited fasting hyperglycemia and largely abolished the reduction of gastric emptying and absorption caused by BNP infusion [13]. A number of prospective studies have suggested that obesity and type-2 diabetes (T2D) promote a state of relative NPs deficiency, so-called “natriuretic handicap” [14,15]. This observation leads to the hypothesis that NP deficiency contributes to the susceptibility of individuals with metabolic disorders to hypertension. In addition, for “NPs excess” in women compared with men [16], a large number of works have explored gender differences in the physiological functions of NPs/NPRs, which states a stimulating effect of estrogens on the NPs/NPRs [17,18], but androgens do the opposite [19,20,21]. Using ovariectomized and estrogen-treated animals and population-based studies, the researchers found that estrogen may have a direct effect on cardiac NP gene expression and release through ovarian hormones or through mediating mechanisms such as RAAS. Then, NP antagonizes the action of angiotensin II (Ang II) on vascular tone, inhibits renin and aldosterone secretion, renal tubule sodium re-absorption, and vascular cell growth. So, to some extent, elevated NP levels in women may be clinically relevant to sex differences in the relative risk of developing metabolic and cardiovascular diseases [22].

Barorereflex keeps blood pressure (BP), heart rate, and blood volume within a narrow physiological range. Baroreceptors are embedded in the walls of arteries, veins, and the heart, and they continuously signal through the vagus (nodose ganglion (NG)) and glossopharyngeal nerve to the nucleus tractus solitarius (NTS) located in the brainstem. When blood pressure, blood volume, or both rise, baroreceptors are activated by stretching [23]. From the NTS, there is an inhibitory pathway that restrains sympathetic outflow to the vasculature. Through interneurons in the caudal ventrolateral medulla (CVLM), neurons in the NTS inhibit sympathetic (premotor) pacemaker neurons in the rostral ventrolateral medulla (RVLM). Axons of barosensitive neurons in the RVLM descend through the spinal cord and activate sympathetic efferent pathway to skeletal muscle and mesenteric and renal vessels. Moreover, NTS projects directly to the preganglionic parasympathetic nerves of the nucleus ambiguus (NA) in the medulla oblongata, activating vagal efferents to slow heart rate and activating postganglionic parasympathetic neurons to the sinoatrial node [23]. With the development and progression of cardiovascular diseases, changes in barorereflex sensitivity (BRS) contribute to the reciprocal decrease in parasympathetic activity and the increase in sympathetic activity [24]. Damaged BRS is commonly observed in hypertension combined with various metabolic disorders [25], and sexual dimorphism in baroreflex afferent function has been well documented [26,27]. The widely distribution of NPs and the receptor-mediated brain-to-blood efflux transport system of blood-brain barrier has strongly suggested that these peptides, besides their well-defined peripheral actions, may play a role in central cardiovascular regulation [28], but whether it takes part in neuroregulation of BP through the baroreflex afferent pathway remains to be explored.

Therefore, we used the ovariectomy (OVX) rats [29] and high-fructose-drinking-induced hypertension (HFD) rats [30] as estrogen-deficiency and metabolic hypertensive model, respectively, compared against the normal control group to investigate the effects of NPs on BP modulation, BRS and the changes in expression profiles of NPs/NPRs in the NG and NTS under normotensive and hypertensive conditions. These results have demonstrated that NPs/NPRs are novel targets for neuroregulation of BP via baroreflex afferent pathway.

## 2. Results

### 2.1. Natriuretic Peptides-Mediated BP Reduction by NG Microinjection under Physiological Condition

**Figure 1:**
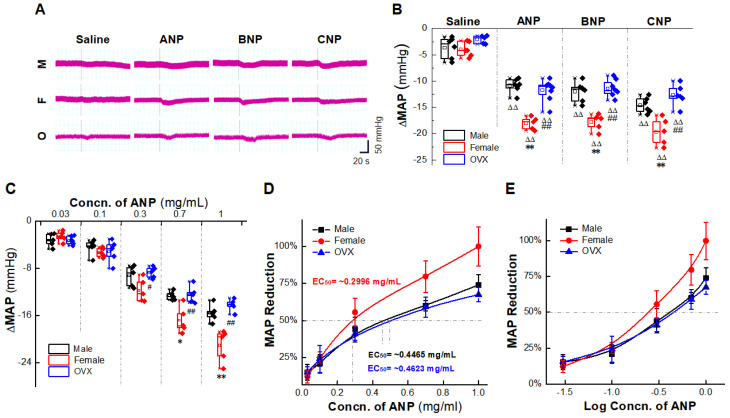

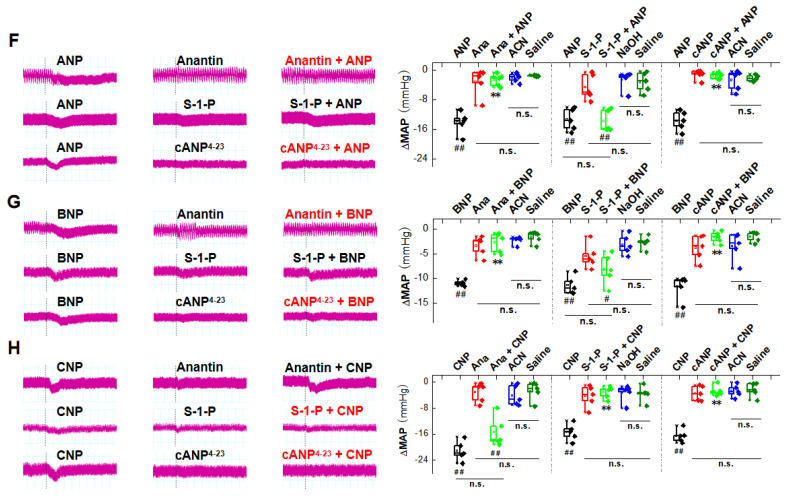
Natriuretic peptides (NPs)—mediated blood pressure (BP) reduction by nodose ganglion (NG) microinjection under physiological condition. (**A**) Real-time monitoring of mean arterial pressure (MAP) changes with the dotted line as the starting point of administration (ANP 1 mg/mL, saline solution, 3 µL). (**B**) Quantitative analysis of BP changes (the difference between nadir after NPs injection and initial MAP). Averaged data was presented as mean ± SD. ** *p* < 0.01 vs. male rats; ^##^ *p* < 0.01 vs. female rats; ^ΔΔ^ *p* < 0.01 vs. saline-injection control rats; *n* = 5–8, from 6 rats for male and female group, and 5 rats for the OVX group. (**C**) Effects of different concentrations of ANP (1 mg/mL, 3 µL) microinjection in the male rats. Averaged data was presented as mean ± SD. * *p* < 0.05, ** *p* < 0.01 vs. male rats, ^#^ *p* < 0.05, ^##^ *p* < 0.01 vs. female rats, *n* = 5 rats for each group. (**D**,**E**) Dose-response curve of ANP microinjection. EC_50_ values were calculated in male, female, and ovariectomized (OVX) rats. Averaged data were presented as mean ± SD. (**F**–**H**) NG microinjection was performed in male group. ANP/BNP/CNP 1 mg/mL, saline solution; anantin 1 mg/mL, ACN solution; cANP^4–23^ 1 mg/mL, ACN solution; S-1-P 4 mg/mL, NaOH solution; ACN 0.2 mg/mL, saline solution; and NaOH 0.3 mmol/L, saline solution; 3 µL, respectively. Effects of NPRA inhibitor: Anantin, NPRB inhibitor: S-1-P, and NPRC activator: cANP^4–23^ on the functions of ANP, BNP, and CNP, respectively. The representative bands of MAP fluctuation monitored in real time were shown on the left. Summarized data of microinjection was shown on the right. Averaged data was presented as mean ± SD. ** *p* < 0.01 vs. NPs-injection group, ^#^ *p* < 0.05, ^##^ *p* < 0.01 vs. saline-injection control group, n.s., no significant difference. *n* = 5 rats for each group.

We conduct NG microinjection because the effects of NPs on BP regulation through peripheral circulation have been identified, but whether NPs can induce direct BP reduction via baroreflex afferent pathway still needs our immediate attention. Therefore, we selected normal male, female, and OXV rats (4 weeks after surgery) (Section 4.4) to conduct NG microinjection. After OVX models were established, their systolic blood pressure (SBP) increased gradually, presented significantly higher than the female rats from the second week (Section 4.3) (Figure S1A). Results of BP monitoring (Section 4.6) revealed that the mean arterial pressure (MAP) of female rats was significantly lower than the other two groups before injection (Figure S1B). After NPs (1 mg/mL) were microinjected directly into the NG, the MAP was significantly reduced in all tested groups, while small fluctuations were observed after saline injection (solvent control) (Figure 1A). The normalized data showed that a more potent BP reduction (ΔMAP) was observed in female rats than male and OVX groups (Figure 1B). Similar trends appeared on ANP microinjection of concentration gradient which indicated a dose-dependent manner (Figure 1C). The concentration for 50% of maximal effect (EC_50_) for BP reduction in the presence of ANP in OVX group was approximately 1.5 times that in the female group (Figure 1D–E).

In order to test the potential involvement of NPRs in NPs-mediated BP reduction, the similar experiments were also carried out with anantin (1 mg/mL, acetonitrile (ACN) solution, NPRA inhibitor) [31], sphingosine 1-phosphate (S-1-P, 4 mg/mL, NaOH solution, NPRB inhibitor) [32], and ring-deleted ANP analogue (cANP^4–23^,1 mg/mL, ACN solution, NPRC activator) [33] before NG microinjection of NPs. As expected, these inhibitors/activators did not induce a significant BP drop, but abolished the effect of NPs on MAP reduction with different affinity (Figure 1F–H). Anantin significantly inhibited the BP reducing effect of ANP and BNP, S-1-P had a similar inhibitory effect on the effect of CNP, and cANP^4–23^ produced the same inhibitory effect on three kinds of NPs. These results suggest that the anti-hypertensive effects of NPs through NG microinjection are directly mediated by binding to the corresponding NPRs with different receptor affinities. ANP and BNP prefer to bind to NPRA, while CNP prefers to bind to NPRB, and NPRC has strong affinities with all three types of NPs.

### 2.2. Gender-Differentiated Distribution of NPRs in Adult Male, Age-Matched Female, and Ovariectomized Female Rats

To further explore the relationship between NPs-mediated gender-related BP reduction and NPRs expression in baroreflex afferent pathway, molecular biology approaches were carried out to detect the expression of NPRs in both NG and NTS sites.

**Figure 2:**
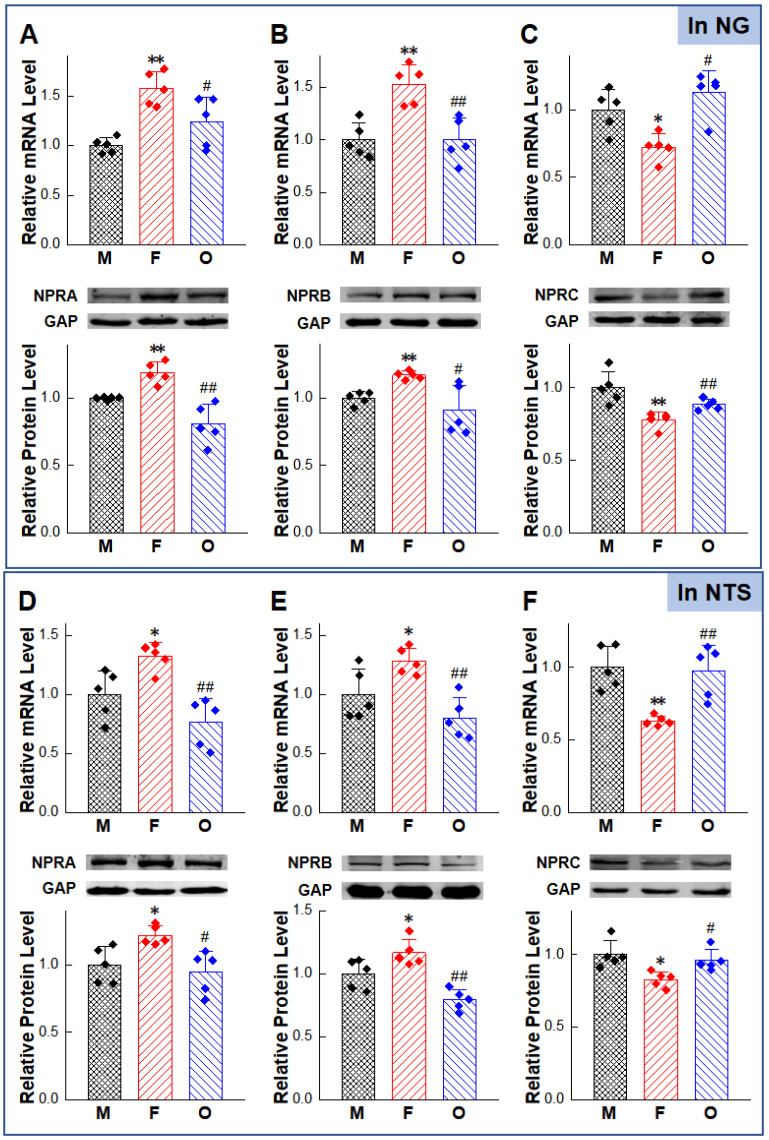

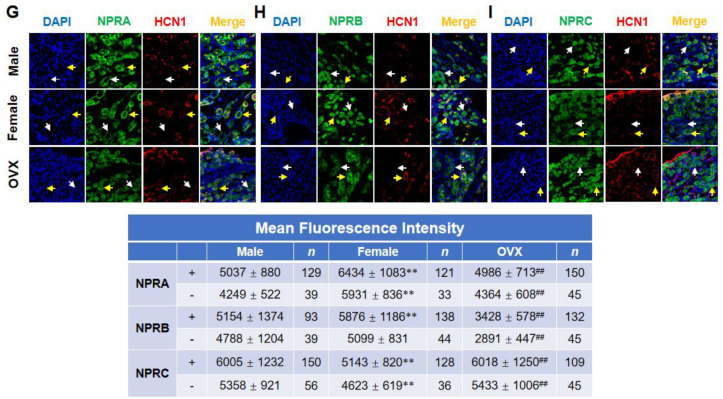
Gender-related differential expression of natriuretic peptide receptors (NPRs) in the nodose ganglia (NG) and nucleus tractus solitarius (NTS). (**A**–**C**) mRNA and protein expression of NPRs in NG of adult male, age-matched female, and OVX rats. * *p* < 0.05, ** *p* < 0.01 vs. male rats, ^#^ *p* < 0.05, ^##^ *p* < 0.01 vs. female rats, *n* = 5. (**D**–**F**) mRNA and protein expression of NPRs in NTS of adult male, age-matched female and OVX rats. * *p* < 0.05, ** *p* < 0.01 vs. male rats, ^#^ *p* < 0.05, ^##^ *p* < 0.01 vs. female rats, *n* = 5. (**G**–**I**) Representative immunostaining for NPRA, NPRB, and NPRC; the hyperpolarization-activated channel specifically expressed on myelinated afferents (HCN1-positive) and neurons were labeled by the antibodies against DAPI (blue), HCN1 (red), and NPRs (green). The yellow arrows pointed to HCN1 positive cells, and the white arrows pointed to HCN1 negative cells. Magnification power was 200×. The bottom table: Quantization results of immunohistochemical staining of NPRs. Data was shown as mean ± SD. ** *p* < 0.01 vs. male rats, ^##^ *p* < 0.01 vs. female rats, from 4–5 slices.

We sampled NG and NTS tissues (Section 4.8). The mRNA expression levels (Section 4.9) of NPRA and NPRB in female rats were higher than male and OVX rats. The expression level of NPRC showed the opposite trend, namely, the expression level of NPRC mRNA in female rats was lower than male and OVX rats. The protein detection (Section 4.10) results also showed the same trend as the PCR results (Figure 2A–F). In addition, we also used immunofluorescence technology (Section 4.11) to explore the cellular distribution of NPRs in myelinated (HCN1-positive) and unmyelinated (HCN1-negative) NG neurons (Figure 2G–I). Results showed that NPRA, NPRB, and NPRC were expressed in the membrane and cytoplasm in both myelinated (HCN1-positive) neurons and non-myelinated (HCN1-negative) neurons in male, female, and OVX rats. Quantitative analysis of fluorescence intensity (Figure 2 Bottom table) showed the same trend of expression level as Western blot.

### 2.3. Anti-Hypertensive Effects of NPs on Hypertensive Condition of HFD Rat Models

**Figure 3:**
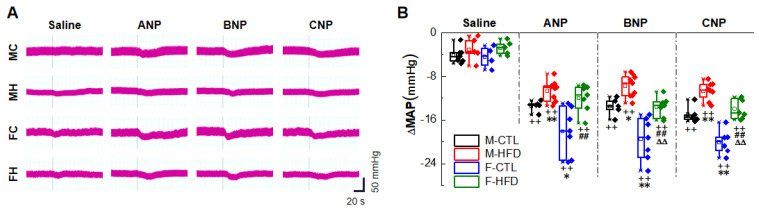
Blood pressure (BP) reduction effects of natriuretic peptides (NPs) under hypertensive condition of high-fructose-drinking-induced hypertension (HFD) rat models. (**A**) Real-time monitoring of BP changes (NPs 1 mg/mL, 3 µL). The representative recordings of mean arterial pressure (MAP) were collected from male control rats (MC), male-HFD rats (MH), female control rats (FC), female-HFD rats (FH). (**B**) Quantitative analysis of changes in MAP. Averaged data was presented as mean ± SD. * *p* < 0.05, ** *p* < 0.01 vs. M-CTL with the same treatment, ^##^ *p* < 0.01 vs. F-CTL with the same treatment, ^∆∆^ *p* < 0.01 vs. M-HFD with the same treatment, ^++^ *p* < 0.01 vs. saline-injection control group, *n* = 5–11, from 5 rats for M-HFD and F-HFD group, 6 rats for M-CTL group, and 7 rats for F-CTL group.

Metabolic disorders and hypertension are frequently co-existed due to similar risk factors, so we used HFD rats (4 weeks after high fructose drinking) (Section 4.5) to mimic hypertension with metabolic abnormalities and carried out the following experiments. After high fructose drinking, the SBP of rats of both genders was increased, with significant differences from the control group of the same sex (Section 4.3) (Figure S2A). The MAP (Method-4.6) before NG microinjection showed the same trend with SBP (Figure S2B). NPs reduced BP in HFD rats through NG microinjection, and the effects also had gender difference, but their antihypertensive effects were not as obvious as those of the control group of the same sex (Figure 3A,B).

### 2.4. BRS Enhancement Effect of NPs Intravenous Injection

**Figure 4:**
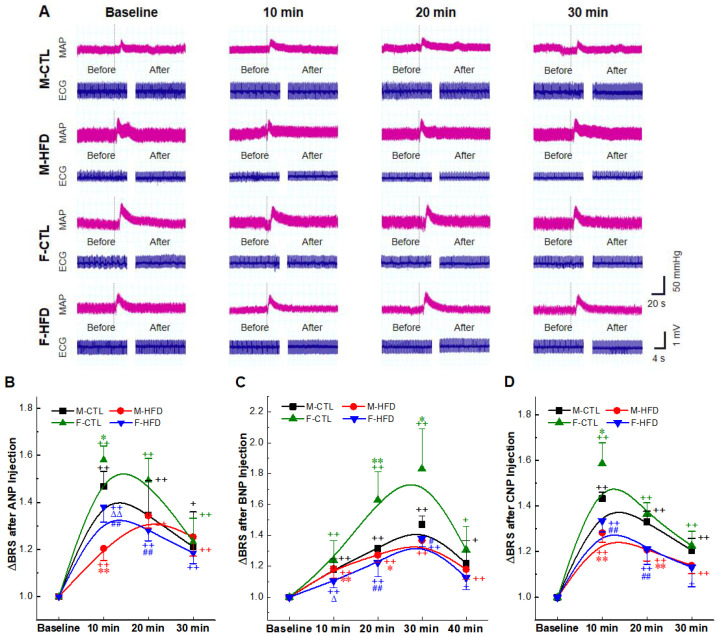
The baroreceptor sensitivity (BRS) improvement effects of natriuretic peptides (NPs) administration in high-fructose-drinking-induced hypertension (HFD) rats. NPs 1 mg/mL, 25 µg/kg was administrated intravenously (i.v.). (**A**) The mean arterial pressure (MAP) and heart rate (HR) were recorded in the presence of ANP before and after intravenous injection of 3 µg/kg phenylephrine (PE). The BRS was presented as ΔHR/ΔMAP (bpm/mmHg). (**B**–**D**) Quantitative analysis results of BRS. Averaged data was presented as mean ± SD. * *p* < 0.05, ** *p* < 0.01 vs. M-CTL with the same treatment, ^#^ *p* < 0.05, ^##^ *p* < 0.01 vs. F-CTL with the same treatment, ^∆^ *p* < 0.05, ^∆∆^ *p* < 0.01 vs. M-HFD with the same treatment, ^+^ *p* < 0.05, ^++^ *p* < 0.01 vs. baseline, *n* = 5 rats for each group.

We further tested BRS to explore whether NPs intravenous (i.v.) injection can affect the function of baroreflex. Changes in BP were monitored by femoral artery cannula, and the vasoactive drug phenylephrine (PE3, 3 µg/kg) was administered by femoral vein; the initial BRS before NPs administration and the BRS after administration were recorded for comparison (Section 4.7) (Figure 4A). Intriguingly, the aberrantly decreased BRS in HFD rats (Figure S3) was significantly reversed by NPs i.v. infusion (Figure 4B–D) in a time-dependent manner. This enhancement of BRS was also found in the control rats, and it was more obvious than that in the HFD rats. The peak of effect of BNP appeared at 30 min, while that of ANP and CNP appeared at about 10 min. Different peak time may be related to different metabolic half-lives of NPs.

### 2.5. Abnormal Expression of NPs/NPRs in the NG and NTS of HFD Rat Models

**Figure 5:**
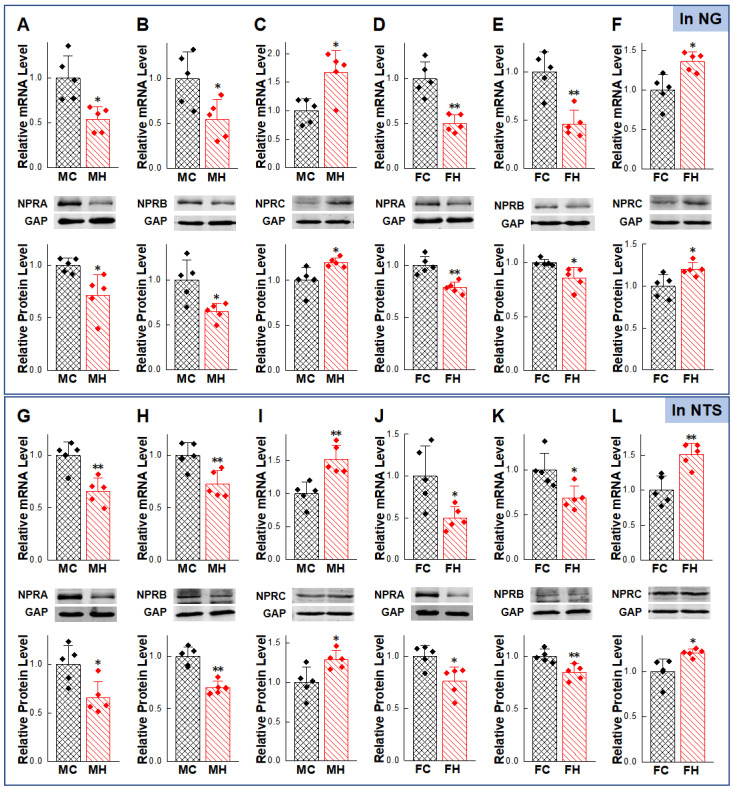
Abnormal expression of natriuretic peptides receptors (NPRs) in nodose ganglion (NG) and nucleus tractus solitarius (NTS) under hypertensive condition. Expression changes in NPRs were verified in NG (**A**–**F**) and NTS (**G**–**L**) of high-fructose-drinking-induced hypertension (HFD) models of adult male and female rats. Averaged data was presented as mean ± SD. GAPDH means the internal control. * *p* < 0.05, ** *p* < 0.01 vs. male or female control rats, *n* = 5.

**Figure 6:**
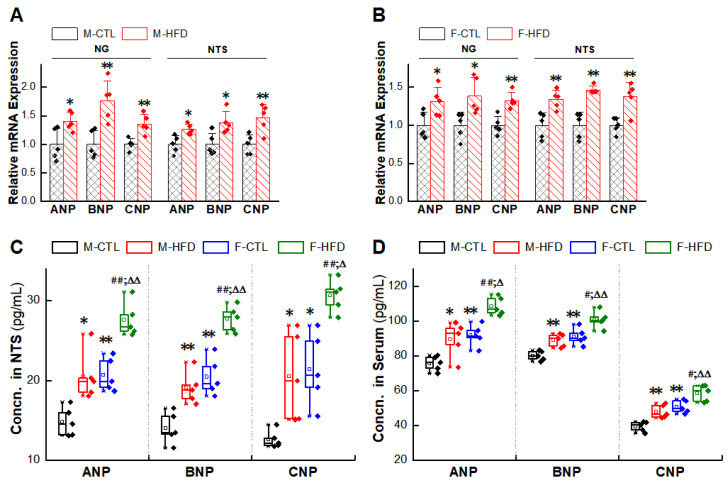
Upregulation of expressions of natriuretic peptides (NPs) in nodose ganglion (NG) and nucleus tractus solitarius (NTS) of high-fructose-drinking-induced hypertension (HFD) rat models. (**A**,**B**) mRNA expression of NPs. Averaged data was presented as mean ± SD. GAPDH means the internal control. * *p* < 0.05, ** *p* < 0.01 vs. male or female control rats, *n* = 5. (**C**,**D**) Concentrations of NPs measured with ELISA assay. Averaged data was presented as mean ± SD. * *p* < 0.05, ** *p* < 0.01 vs. M-CTL, ^##^ *p* < 0.01 vs. F-CTL, ^∆^ *p* < 0.05, ^∆∆^ *p* < 0.01 vs. M-HFD, *n* = 5.

Based upon elevated BP, reduced BRS in HFD rats, and anti-hypertensive effects of NPs, the expression levels of NPRs in NG/NTS were detected (Section 4.9 and Section 4.10). The mRNA expressions of NPRA and NPRB in HFD rats were down-regulated, while the expression of NPRC was up-regulated in the HFD rats, which was higher than those in the control group of the same sex. The protein results also showed the same trend as the PCR results (Figure 5A–L). Therefore, we hypothesized that the impaired NPRs expression was a likely reason for the weaker cardiovascular protection of exogenous NPs in the HFD rats. However, the deficiency of NPs expression was not detected in the HFD group as expected; instead, there was compensatory or pathologic increase in NPs expression (Section 4.9 and Section 4.12) (Figure 6A–D).

## 3. Discussion

**Figure 7:**
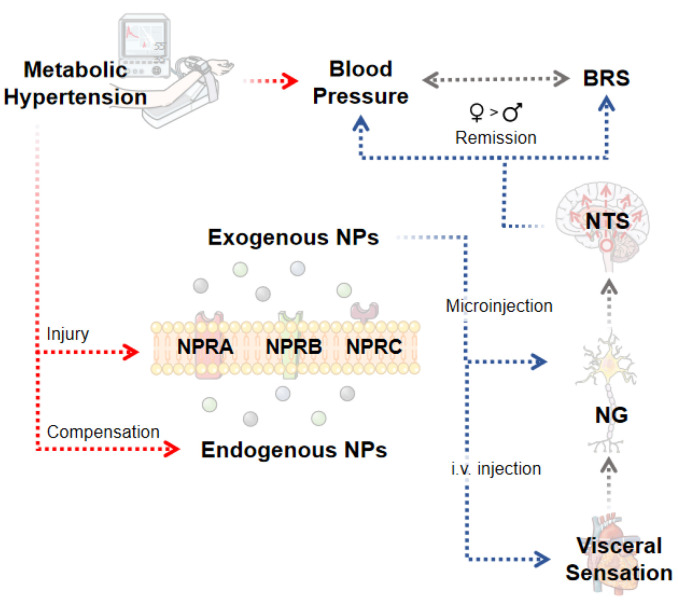
Schematic diagram explaining natriuretic peptides (NPs)—mediated anti-hypertensive effects via baroreflex afferent pathway. This study confirmed that microinjection of NPs in the nodose ganglion (NG) can lower blood pressure under both physiological and pathophysiological status. Meanwhile, exogenous NPs can perform baroreceptor sensitivity (BRS) improvement effect. These anti-hypertensive functions and the expression of natriuretic peptide receptors (NPRs) were closely related to estrogen. Abnormal expression of NPs and its receptors in nodose ganglion (NG) and nucleus tractus solitarius (NTS) in models of hypertension with metabolic disorders suggests a negative feedback regulation.

Our investigation has demonstrated for the first time that direct microinjection of NG by NPs reduces BP, and i.v. administration of NPs enhances BRS. These two cardiovascular protective effects exist in the physiological state and the pathophysiological condition of metabolic hypertension (HFD rats). In addition, these effects were changed in an estrogen-dependent manner and were closely linked to the differential expression of NPRs in baroreflex afferent pathway. Intriguingly, pathologic up-regulation of endogenous NPs in the NG/NTS in HFD rats indicated that there was a potential negative feedback regulation of NPs/NPRs in the baroreflex afferent pathway (Figure 7) to compensate increased BP. In this study, the cardiovascular protective effects of NPs/NPRs have been linked with the baroreflex afferent function and contributed robustly to autonomic control of BP regulation, proving that NPs and their receptors are novel targets for neuroregulation of BP.

The potential roles of NPs in modulating physiological functions of the central nervous system (CNS) have been a hotspot for the wide distribution of all three NPs in the brain [34]. Microinjection of exogenous ANP into the caudal NTS has been shown to produce a significant decrease in BP and increase the firing rate of NTS neurons, suggesting that ANP induced activation of NTS neurons may mediate depressor responses [35,36]. In SHR rats, inhibiting the actions of endogenous ANP in the NTS further elevates BP, suggesting that the peptide is responsible for in the regulation of cardiovascular baroreceptor signal to this region of the brain [37]. In order to complete its direct role in the baroreflex afferent pathway, we conducted the experiment of NG microinjection. Consistent with the expected results, NPs directly reduced BP in a dose-dependent manner (Figure 1). These data indicate that NG is a potential target for NPs action on neurocontrol of BP regulation.

The natriuretic peptides exert their effects through interaction with high-affinity receptors on the surface of target cells. NPRA and NPRB are linked to the cGMP-dependent signaling cascade and mediate many of the cardiovascular and renal effects of the natriuretic peptides. NPRC is involved in clearance of the peptides [38]. Through immunofluorescence technology, we confirmed the expressions of NPRs in NG neurons (Figure 2), in order to clarify the relationship between NPRs in NPs anti-hypertensive effect, we used three kinds of NPRs agonists/inhibitors to explore its inhibitory effect on NPs antihypertensive effect, the results showed that Anantin significantly inhibited the BP reducing effect of ANP and BNP, S-1-P had a similar inhibitory effect on the effect of CNP, and cANP^4–23^ had a similar inhibitory effect on three kinds of NPs (Figure 1). This is consistent with previous reports on the affinity of NPs and NPRs [39], namely, ANP/BNP mainly combines with NPRA, and CNP mainly combines with NPRB to produce anti-hypertensive effect. NPRC abolished the anti-hypertensive effect of all kinds of NPs in NG. 

On the other hand, the BP reducing effect revealed non-negligible gender difference (Figure 1). The anti-hypertensive effect of the female group was superior to that of the male and OVX group with the lowest EC_50_ value. Recent literatures from our laboratory can provide an explanation for this phenomenon, which show that besides the widely recognized A-type and C-type BRNs, adult female rats express a novel kind of female-specific subpopulation of low-threshold myelinated Ah-type BRNs [40,41], which present not only in NG, but also in NTS [42]. These neurons are distributed in male and female newborn rats without gender difference. However, Ah-type BRNs are rarely found in adult male rats and exhibited an excitability profile which is strongly influenced by circulating sex hormones [43]. Due to its unique electrophysiological characteristics (low action potential firing threshold, fast upstroke velocity, and narrow action potential duration), Ah-type BRNs are relatively active, namely, small arterial blood pressure fluctuations can stimulate Ah-type BRNs to generate action potential and perform their functions in blood pressure regulation [40]. The presence of this female-specific neuron further confirms the important role of the baroreflex afferent pathway in the gender difference of BP regulation. Meanwhile, our previous studies found that various circulatory factors associated with the modulation of BP could also act on the baroreflex system. The expressions of fibroblast growth factor-21 (FGF-21) receptors [44], Neuropeptide Y (NPY) [45] and Ang-Ⅱ receptors [46], were found in NG and NTS and presented apparent sexual dimorphism. This reminds us to detect the expression of NPRs in NG/NTS and the gender-specific expression was found (Figure 2), which may be one of the reasons for the gender difference in the anti-hypertensive effect of NG microinjection of NPs; this ties NPs/NPRs and gender differences neuroregulation compactly.

Due to the crucial role of NPs in the regulation of water and salt metabolism, we would like to pick a typical metabolic hypertension rat model for functional experiments. Changes in diet with increasing fructose consumption have been studied extensively as a contributing factor to the development of metabolic diseases and associated hypertension [47,48], so we conducted HFD model rats to detect the BP regulatory function of NPs under hypertensive state. Sugar added to numerous manufactured food products, in the form of sucrose or high-fructose corn syrup, both of which are composed of nearly equal amounts of glucose and fructose, Whereas the glucose drink has no effect on blood pressure, fructose consumption has an acute effect on rising BP [49], making high-fructose-drinking rats become a classic model [50]. Studies to date show that the mechanisms by which excess fructose increases BP into three broad categories: increased salt absorption, endothelial dysfunction, and chronic stimulation of the sympathetic nervous system [51]. Moreover, there is reversible impaired baroreflex function in HFD rats [44]. These reports remind us there is an underlying link between NPs and neuroregulation. To further investigate whether the baroreflex afferent pathway is involved in the BP neuromodulation of NPs in HFD, we conducted the similar NG administration experiments in hypertensive rats and obtained a conclusion of anti-hypertensive effect of NPs (Figure 3).

Though stellate ganglion injection therapy has been used in PTSD treatment [52], nodose ganglion injection therapy has not been adapted in clinical, and the only approved therapeutic application of the NPs is still intravenous treatment [53,54,55]; therefore, we investigated the influence of baroreflex function after intravenous injection, and found that BRS was enhanced by NPs in hypertensive rats (Figure 4). However, the improvement effects in the hypertension group were not as superior as that in the control group, possibly because of the impaired receptor expression (Figure 5). In addition, we found that three kinds of NPs had distinct peak action time, which may be determined by different blood half-lives. As reported, the half-life of ANP in human plasma is approximately 2 min [56], which is comparable to that of CNP [57], whereas BNP has a longer half-life, being approximately 20 min [58]. In addition, contrary to our expectation, endogenous NPs expression levels were up-regulated in NG and NTS tissues in the hypertensive group (Figure 6). In combination with the anti-hypertensive effect of NPs and the improvement effects of baroreflex, we speculated that there might be a negative feedback regulation in the baroreflex pathway. Hypertension results in elevated BP, impaired BRS, and up-regulation of endogenous NPs expression. Long-term stimulation of increased NPs expression leads to impaired NPRs expression in the NG and NTS and forming the NPs BP neurocontrol loop.

Of course, there are still some remaining questions about the BP neuroregulatory mechanisms of NPs. In addition to the role of the NPRs, the post-receptor loop also possesses dramatic significance, such as the NPRs-PKG pathways and the interactions between NPs and other BP regulatory factors (such as Ang-Ⅱ, glutamate, endothelial nitric oxide synthase (eNOS), and so on). Other components of the barorereflex pathway, such as NA, CVLM, and RVLM, also deserve attention in the regulation of BP by NPs. Whether there are interactions between other endocrine processes and neuromodulation in the regulation of metabolic hypertension by NPs should also be investigated in the future. In addition, due to the gender-specific distribution of Ah-type neurons, electrophysiology research is imperative. Single baroreceptor neurons (BRNs) can be achieved via whole-cell patch clamp technique and single-cell qRT-PCR technology can be conducted to explore the distinct expressions of NPRs in A-/Ah-/C-type neurons and whether NPs can alter electrophysiological parameters of BRNs. To clarify these neuroregulatory roles of NPs in BP not only provides a new explanation for gender differences in BP, but also provides a new idea for the pathogenesis of metabolic hypertension and a novel target and strategy for clinical management of hypertension, which would produce a profound clinical impact.

## 4. Materials and Methods

### 4.1. Animals

Age-matched sexually mature male and female Sprague-Dawley (SD) rats weighting 200–220 g were purchased from the experimental animal center of the Second Affiliated Hospital of Harbin Medical University (Harbin, China). Animal Certificate No.: SCXK 2019-001. All animal protocols were pre-approved by the Institutional Animal Care and Use Committee at Harbin Medical University, which are in accordance with the recommendations of the Panel on Euthanasia of the American Veterinary Medical Association and the National Institutes of Health publication “Guide for the Care and Use of Laboratory Animals” (https://www.nap.edu/readingroom/books/labrats/, accessed on 1 September 2017).

### 4.2. Chemicals

The chemicals used in the experiments were supplied by their manufacturers as follows (Table S1).

### 4.3. Systolic Blood Pressure Measurements

The non-invasive SBP (mmHg) was measured weekly in conscious rats with a manometer-tachometer (BP-2010E, Softeron Biotechnology, Beijing, China) using the tail-cuff method. Rats were placed in a plastic holder under a 37 °C temperature-controlled quiet environment, and the measurements were taken at the same time of the day (8:00–12:00 a.m.) to minimize the influence of circadian cycles on BP. The average value of SBP for each rat was obtained from over five SBP readings after the rats were acclimated to the environment.

### 4.4. Surgical Procedures of Ovariectomy

The surgery was performed following protocols described in detail previously [59]. The animals were anaesthetized (3% amobarbital sodium, 0.3 mL per 200 g, i.p) and placed in a lateral position. After both flanks were shaved and disinfected with 70% ethanol and povidone iodine (7.5%), an approximate 2.0 cm incision was made on the left lateral side along a line spanning from 2nd to 5th lumbar vertebra to expose the ovaries. The left ovary and associated fat were located and externalized by gentle retraction. After removing the ovary, the peritoneal cavity, muscle layers, and skin were sutured successively with 4.0 absorbable sutures and disinfected with iodophor. The same procedure was repeated to remove the right ovary. Finally, penicillin (80,000 Units) was given via intramuscular injection. After recovering from anesthesia, the animals were monitored for at least 30 min to ensure that there was no bleeding from the surgery.

### 4.5. High Fructose-Drinking Induced Hypertension Model

Rats were randomly housed in groups of 5 rats per cage in a controlled environment (25 °C with 12 h light/dark cycles) and divided into two experimental groups after one week of adaptation. Rats received standard rat chow and tap water (control group, CTL), or water containing 10% (*w*/*v*) fructose (Zhiyuan Chemical Co., Binzhou, China) for 4 weeks. The SBP of rats for each group was recorded weekly.

### 4.6. Nodose Ganglion Microinjection

As described in our previous experiments [46], anaesthetized rats were placed in a supine position, and the femoral arterial was cannulated under a stereo microscope (Olympus, Japan, 0.8×, 10×) connecting the physiological pressure transducer (AD Instruments MLT 844, Norway) in order to record MAP. Then, a 4.0 cm longitudinal incision was made along midline in the neck to isolate left side nodose ganglion under stereomicroscopy. After the baseline of MAP was stable, 3 µL of NPs, NPRs inhibitors/activators and their solvents were injected into the NG with a precision tailored micro syringe (Hamilton, O.D. × I.D. = 0.31 × 0.16). Finally, the changes in MAP before and after injection were analyzed by the software of Labchart 7 Pro software (AD instruments, Australia).

### 4.7. Acute Intravenous NPs Injections and Baroreflex Sensitivity Detection

After 4 weeks of high fructose-drinking or normal fed, the anesthetized rats were subjected to femoral artery and vein catheters to record MAP, and injects drug, respectively. Heart rate was measured by electrocardiograph (ECG), which was recorded with two needle electrodes placed subcutaneously on the lower left forelimb (+) and the right hind paw (−). Use classic method to measure BRS (ΔHR/ΔMAP, bpm/mmHg)—intravenous injection of vasoactive drug, the α-adrenoreceptor agonist phenylephrine (PE).

After intravenous NPs (25 µg/kg) injections, MAP (mmHg) of the femoral arterial was monitored continuously for 30/40 min. Meanwhile, the BRSs induced by PE (3 µg/kg) were detected before (Baseline of BRS) and after NPs injections (at 10, 20, and 30 min for ANP/CNP, and 10–40 min for BNP) in HFD and control rats.

### 4.8. Tissue Preparation of NG and NTS

Upon lack of reflex response to tail pinch after 3% pentobarbital sodium or 10% chloral hydrate intraperitoneal administration, the rats were immediately sectioned at the end of the upper thoracic segment (including both forelimbs) in order to preserve enough length (2 cm) of the vagus nerve. Under the microscope (Olympus, Japan), the entire NG with the attached nerve trunk was carefully excised and immediately transferred into a Petri dish containing pre-cooled (4 °C) normal saline and then the surrounding connective tissue was gently removed. After rapid freezing in liquid nitrogen, store the tissues in −80 °C refrigerator for future molecular investigation.

For NTS tissue collection, the head of the rat was placed in ventricumbent position to expose the bilateral medulla. The bilateral medulla was trimmed to a 1 cm block (rostral-caudal) centered on the obex under microscope. Cut the NTS nuclei by reference to the stereotactic graph of the rat brain (Paxinos and Watson, the 6th edition, 2007) and freeze it in liquid nitrogen, then transfer to the −80 °C for further examination.

### 4.9. Quantitative Reverse Transcription-Polymerase Chain Reaction (qRT-PCR)

One mRNA sample was extracted from 1–2 (NTS) or 2–3 (NG) rats in the same group. According to the manufacturer’s instructions, the total RNA was extracted by Trizol Reagent and complementary DNA (cDNA) was synthesized with high-capacity cDNA reverse transcription reagent kits (#TYB-FSQ-101, Toyobo, Osaka, Japan). The SYBR Green PCR Master Mix Kit (#24759100, Roche Diagnostics, Basel, Switzerland) was used in qRT-PCR to quantify target genes using Light Cycler^®^ 96 Instrument system (Roche Diagnostics, Basel, Switzerland). The primers for NPs and NPRs (Table S2) were purchased from Invitrogen (Frederick, MD, USA). Data were normalized to GAPDH (internal control) by the 2^−(ΔΔCT)^ comparative method.

### 4.10. Western Blot

The tissue extracted from 1–2 (NTS) or 2–3 (NG) rats in the same group was lysed by lysis buffer (RIPA: SDS: PI, 60:40:1) for 30 min, and the lysate was centrifuged for 15 min to collect supernatant. Protein extracts (100 µg/sample) accessed through a BCA protein assay kit (#P0010S, Beyotime, Shanghai, China) were subjected to 10% SDS-Tris glycine gel electrophoresis and then transferred to a nitrocellulose membrane, which was blocked in 5% defatted milk/phosphate buffered saline buffer for 2.5 h. Then, the membranes were incubated overnight at 4 °C with the primary antibodies (anti-NPRA 1:1000, anti-NPRB 1:100, anti-NPRC 1:2000, anti-GAPDH 1:1000) and incubated with secondary antibodies at room temperature for 55 min. Blots were visualized and quantified by Odyssey infrared imaging system (#ODY-3149, LI-COR, Lincoln, NE, USA) for each group and normalized to GAPDH band intensity. The final results were expressed as fold changes. Antibodies used in this experiment were listed in Table S3.

### 4.11. Immunohistochemical Analysis

The immunohistochemistry protocol for NG was described in our previous reports [46]. For the immunohistochemical staining, the tissues were fixed with 4% buffered paraformaldehyde and cut into the thickness of 10 µm. Penetrating solution (10% BSA and 3% Triton-X in PBS) and goat serum were used to penetrate and block the histological sections at room temperature for 2 h. Then the histological sections were incubated at 4 °C overnight with the primary antibodies of NPRA (1:200), NPRB (1:5) and NPRC (1:200) placed in PBS (20% BSA, 10% goat serum and 6‰ antibody of HCN1 in PBS) overnight at 4 °C. The antibody against HCN1 was used as fluorescent marker to distinguish myelinated and unmyelinated neurons. Appropriate secondary antibodies (1:200) placed in PBS (20% BSA and 10% goat serum in PBS) were incubated for 1 h at room temperature. DAPI (DAPI: PBS, 1:30) was used to stain the nuclei at room temperature for 30 min. Immunofluorescence was observed by fluorescence microscopy. Antibodies used in this experiment were listed in Table S3.

### 4.12. Enzyme Linked Immunosorbent Assay (ELISA)

The concentrations of ANP, BNP, and CNP in NTS were measured by using ELISA kits (E-EL-R0017c for ANP, E-EL-R0126c for BNP, and E-EL-R0284 for CNP, respectively; Elabscience, Wu Han, China).

### 4.13. Statistical Analysis

Excel (Microsoft), SPSS Statistics (IBM) and Origin (OriginLab) were used for statistical analysis and data graphing. Normal distribution was tested by SPSS, and the kurtosis and skewness values were used to calculate corresponding Z-score. For samples that did not conform to the normal distribution, we use the Mann-Whitney U test. For samples conformed to the normal distribution, ANOVA (Tukey’s test) was used to compare the difference among 3 or more tested groups. Student’s *t*-test was used as post-hoc test to identify the significance. Paired *t*-test was used to compare the difference before and after treatment and difference between 2 tested groups. The averaged data was presented as mean ± SD. The criterion for statistical significance was set at *p* < 0.05.

## 5. Conclusions

Our data provide additional evidence showing that NPs plays a crucial role in neurocontrol of BP regulation via baroreflex afferent function and may be potential targets for clinical diagnosis and management of metabolic-related hypertension.

## Data Availability

The data presented in this study are available on request from the corresponding author.

## Supplementary Materials

The following supporting information can be downloaded at: https://www.mdpi.com/article/10.3390/ijms232113619/s1.
