# Supplementary material for: Natriuretic Peptides—New Targets for Neurocontrol of Blood Pressure via Baroreflex Afferent Pathway
Source: Int J Mol Sci. 2022 Nov 7;23(21):13619. doi: 10.3390/ijms232113619 (PMC9657840; doi:10.3390/ijms232113619)

Online only Supplemental Materials

Natriuretic Peptides – New Targets for Neurocontrol of Blood Pressure via Baroreflex Afferent Pathway

Li Xin-Yu†, Cui Ya-Li†, Zhang Qing†, Li Qing-Yuan, Cheng Meng-Xing, Sun Jie, Cui Chang-Peng, Fan Xiong-Xiong, Li Bai-Yan*

[1]Department of Pharmacology (State-Province Key Laboratories of Biomedicine-Pharmaceutics of China, Key Laboratory of Cardiovascular Medicine Research, Ministry of Education), College of Pharmacy, Harbin Medical University, Harbin, China

Short running title: NPs & BRx afferent function

† These authors contributed equally to this work.

Correspondence to Bai-Yan Li, Department of Pharmacology, Harbin Medical University, 157 Bao-Jian Rd, Harbin 150081, China; Ph/Fax: 86 451-8667-1354, E-mail: liby@ems.hrbmu.edu.cn.

This PDF file includes:

Tables. S1 to S3 and Figures. S1 to S3

Supplemental Tables

Table S1: Chemicals used in this experiment.

Chemicals	Source	Catalog #
ANP	NJ Peptide Biomart	Pep-06338
BNP	Cayman	24541
CNP	NJ Peptide Biomart	Pep-07437
Anantin	NJ Peptide Biomart	Pep-06271
Sphingosine-1-phosphate	Cayman	62570
$cANP^{4-23}$	NJ Peptide Biomart	Pep-06340
ACN	FuYu Chemical	75-05-8
NaOH	Sigma-Aldrich	S8045
Phenylephrine	Sigma-Aldrich	P1240000
Pentobarbital sodium salt	Sigma-Aldrich	P11011
Chloral hydrate	Aladdin	C104202
RIPA lysis buffer	Beyotime	P0013B
SDS	Beyotime	P0013G
Protease Inhibitor Cocktail	MedChemExpress	HY-K0010
DAPI	Beyotime	C1005
Trizol Reagent	Invitrogen	15596-018

Table S2. The list of all qRT-PCR primers used in this investigation.

Gene List	Primers	Sequences
ANP	Forward Primer	5'-GCCGGTAGAAGATGAGGTCA-3'
	Reverse Primer	5'-GGGCTCCAATCCTGTCAATC-3'
BNP	Forward Primer	5'-CAGAGCTGGGGAAAGAAGAG-3'
	Reverse Primer	5'-GGACCAAGGCCCTACAAAAGA-3'
CNP	Forward Primer	5'-GGAGCCAATCTCAAGGGA-3'
	Reverse Primer	5'-TGCCGCCTTTGTATTTGC-3'
NPRA	Forward Primer	5'-CCTTTCAGGCTGCCAAAAT-3'
	Reverse Primer	5'-ATCCTCCACGGTGAAGTTGA-3'
NPRB	Forward Primer	5'-TCTATGCCAAGAAGCTGTGG-3'
	Reverse Primer	5'-CCAGGCCTTCCAAGTAGAAA-3'
NPRC	Forward Primer	5'-GGACCGCGAAGCCTGAGT-3'
	Reverse Primer	5'-ATGGACACCTGCCCGGCGATACCT-3'
GAPDH	Forward Primer	5'-ATGACTCTACCCACGGCAAG-3'
	Reverse Primer	5'-TACTCAGCACCAGCATCACC-3'

Table S3: Antibodies used in this experiment.

Antibodies	Source	Catalog #	RRID
Rabbit polyclonal anti-NPRA	NOVUS	NBP1-31333	AB_2155457
Mouse polyclonal anti-NPRB	SANTA CRUZ	sc-293451	AB_2847874
Rabbit polyclonal anti-NPRC	NOVUS	NBP1-31365	AB_2155906
Mouse monoclonal anti-GAPDH	ABclonal	ac002	AB_2736879
Rabbit polyclonal anti-HCN1	OriGene	TA328955	AB_2847873
Mouse monoclonal anti-HCN1	OriGene	TA326543	AB_2847872
IRDye® 800CW Goat anti-Rabbit IgG (H + L)	LI-COR	926-32211	AB_621843
IRDye® 800CW Goat anti-Mouse IgG (H + L)	LI-COR	926-32210	AB_621842
Alexa Fluor® 488 goat anti-rabbit lgG (H+L)	Life Technology	A-11034	AB_2576217
Alexa Fluor® 488 goat anti-mouse lgG (H+L)	Life Technology	A-11029	AB_138404
Alexa Fluor® 594 goat anti-rabbit lgG (H+L)	Life Technology	A-11037	AB_2534095
Alexa Fluor® 594 goat anti-mouse lgG (H+L)	Life Technology	A-11032	AB_2534091

Supplemental Figures

Figure S1: Blood pressure (BP) of rats of different gender groups under physiological condition. (A) Changes in rat systolic blood pressure (SBP) after ovariectomy for 4 weeks. Results were analyzed using two-tailed unpaired Student's *t*-test and one-way ANOVA between and among groups, and averaged data was presented as mean ± SD. $^{**}P < 0.01$ *vs*. male rats, $^{\#\#}P < 0.01$ *vs.* female rats, $n = 10$. **(B)** Mean arterial pressure (MAP) of rats before nodose ganglia (NG) microinjection. Averaged data was presented as mean ± SD. $^{**}P < 0.01$ *vs*. male rats(M), $^{\#\#}P < 0.01$ *vs.* female rats (F), from 6 rats for male and female group, and 5 rats for OVX group.

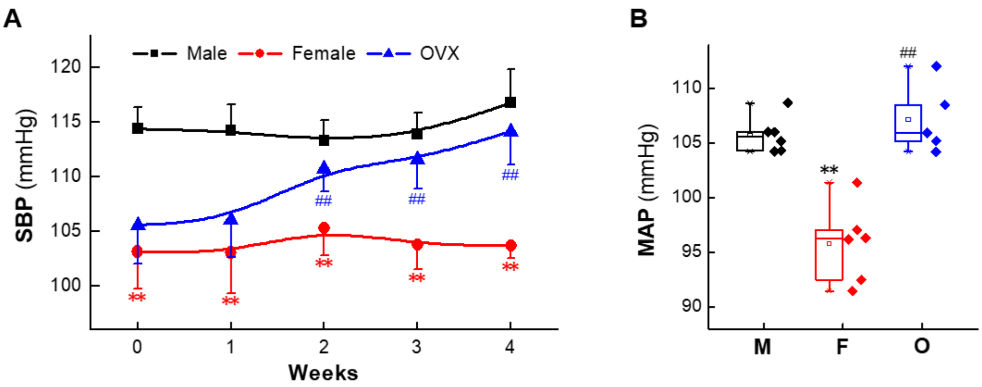

Figure S2: Blood pressure (BP) of rats of different gender groups under pathophysiological condition. (A) Changes in rat systolic blood pressure (SBP) after high fructose-drinking for 4 weeks. Averaged data were presented as mean ± SD. $^{**}P < 0.01$ *vs.* M-CTL, $^{\#\#}P < 0.01$ *vs.* F-CTL, $^{\Delta}P < 0.05$, $^{\Delta\Delta}P < 0.01$ *vs.* M-HFD, $n = 10$. **(B)** Mean arterial pressure (MAP) of rats before nodose ganglia (NG) microinjection. Averaged data was presented as mean ± SD. $^{**}P < 0.01$ *vs.* male-control rats (MC), $^{\#\#}P < 0.01$ *vs.* female-control rats (FC), $^{\Delta\Delta}P < 0.01$ *vs.* male-HFD rats (MH), from 5 rats for each MH and FH group, 6 rats for MC group, and 7 rats for FC group..

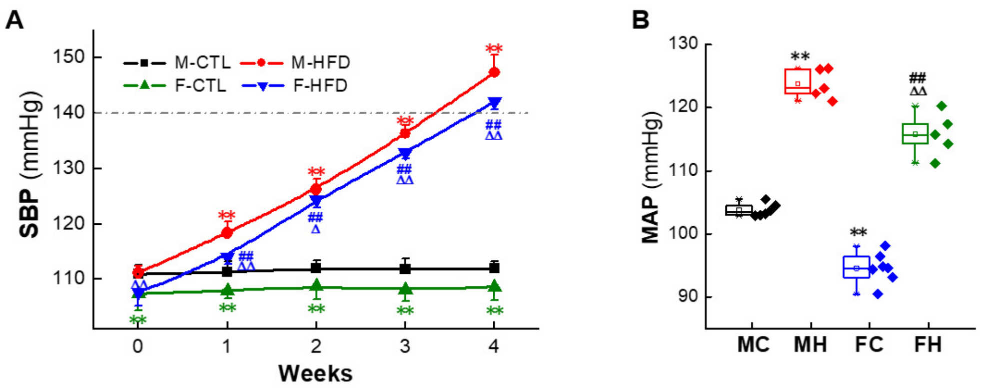

Figure S3: The baroreceptor sensitivity (BRS) baseline before natriuretic peptides (NPs) intravenous injection. Averaged data were presented as mean ± SD. $^{**}P < 0.01$ *vs*. male-control rats (MC), $^{\#\#}P < 0.01$ *vs*. female-control rats (FC), $^{\Delta\Delta}P < 0.01$ *vs*. male-HFD rats (MH), $n = 15$ for each group.

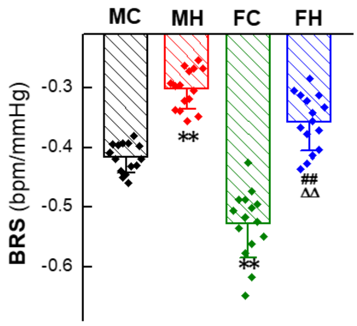

Supplement: Supplementary file 1 [file ijms-23-13619-s001.zip › ijms-1943192-supplementary.pdf]
